# Selenium, Zinc, and Plasma Total Antioxidant Status and the Risk of Colorectal Adenoma and Cancer

**DOI:** 10.3390/metabo14090486

**Published:** 2024-09-06

**Authors:** Miłosława Zowczak-Drabarczyk, Jacek Białecki, Teresa Grzelak, Mikołaj Michalik, Dorota Formanowicz

**Affiliations:** 1 Chair and Department of Medical Chemistry and Laboratory Medicine, Poznan University of Medical Sciences, Rokietnicka 8, 60-806 Poznan, Poland; mzowczakdrabarczyk@ump.edu.pl (M.Z.-D.); doforman@ump.edu.pl (D.F.); 2 Department of General and Minimally Invasive and Trauma Surgery, Franciszek Raszeja Municipal Hospital, Mickiewicza 2, 60-834 Poznan, Poland; jacekt.bialecki@gmail.com; 3 Chair and Department of Physiology, Poznan University of Medical Sciences, Święcickiego 6, 60-781 Poznan, Poland; 4 Omega Family Doctors, Nowy Rynek 6, 62-002 Suchy Las, Poland; mikolaj.michalik@gmail.com

**Keywords:** plasma antioxidant status, selenium, zinc, copper, colorectal cancer, colorectal adenoma

## Abstract

Selenium (Se), zinc (Zn), and copper (Cu) are known to be involved in carcinogenesis and participate in the defence against reactive oxygen species (ROS). This study aimed to evaluate the clinical utility of serum Se, Zn, and Cu concentrations and plasma total antioxidant status (TAS) in the diagnosis of colorectal cancer (CRC) and colorectal adenoma (CRA) in a population of low Se and borderline Zn status. Based on clinical examination and colonoscopy/histopathology, the patients (n = 79) were divided into three groups: colorectal cancer (n = 30), colorectal adenoma (n = 19), and controls (CONTROL, n = 30). The serum Se concentration was lower in the CRC group than in the CRA group (by 9.1%, *p* < 0.0001) and the CONTROL group (by 7.9%, *p* < 0.0001). In turn, the serum Zn concentration was decreased in the CRA group (by 17.9%, *p* = 0.019) when compared to the CONTROL group. Plasma TAS was lower in the CRC group (by 27.8%, *p* = 0.017) than in the CONTROL group. In turn, the serum Zn concentration was decreased in the CRA group when compared to the CONTROL group. Plasma TAS was lower in the CRC group than in the CONTROL group. ROC (receiver operating characteristic) curve analysis revealed that the Se level was of the highest diagnostic utility for the discrimination of the CRC group from both the CRA group (area under ROC curve (AUC) 0.958, sensitivity 84.21%, specificity 100%) and the CONTROL group (AUC 0.873, sensitivity 100%, specificity 66.67%). The Zn and TAS levels were significantly accurate in the differentiation between the groups. An individualised risk of colorectal adenoma and cancer approach could comprise Se, Zn, and TAS assays in the population.

## 1. Introduction

Colorectal cancer (CRC) is one of the most common malignancies worldwide for both sexes. CRC most frequently originates from a precancerous lesion such as an adenomatous polyp, i.e., a colorectal adenoma (CRA) [1,2,3]. Selenium (Se), zinc (Zn), and copper (Cu) are considered to be involved in carcinogenesis, including in the colon, through various mechanisms. All three trace elements indirectly participate in the defence against reactive oxygen species (ROS) [3,4,5]. Selenoproteins mainly mediate the biological functions of Se. Approximately half of all functionally characterised selenoproteins are oxidoreductases (e.g., glutathione peroxidase GPx and thioredoxin reductase TrxR).

Plasma selenoprotein P (SeP) delivers Se to other organs, whereas other selenoproteins are involved in the quality control of protein folding and protein degradation in the endoplasmic reticulum. Moreover, Se influences epigenetic modifications of DNA and histones. Selenium blood status modulates selenoprotein expression in the colon and downstream targets, such as the endoplasmic reticulum stress response, oxidative stress, and inflammatory pathways [5,6]. Pilat et al. discovered that the selenium-rich antioxidant selenoprotein P expression rises as normal colon stem cells transform into adenomas (CRA) that progress into carcinomas (CRC), based on human scRNA-seq datasets [7].

Dietary selenium also affects intestinal microbiota, and, vice versa, gut microorganisms affect the host’s selenium status, selenoproteins expression, and other molecules linked to WNT/β-catenin signalling pathways, influencing the regulation of oxidative stress, inflammation, immune response, apoptosis, and cancer risk [8]. It was found that in colon cancer cells, the TRIM32 gene was overexpressed and linked to a worse prognosis, whereas Se treatment significantly inhibited cell proliferation and migration and stimulated apoptosis [9].

Therefore, Se can be essential in colonic epithelial cells’ response to microbial and oxidative exposure and toxins. A combination of low Se status and single nucleotide polymorphisms (SNPs) in selenoprotein genes can impair their functions and increase the risk of colonic neoplasia [6]. Studies on animal and cellular models provided compelling evidence that Se protects against experimentally induced carcinogenesis in the colon [6]. Research has shown that low levels of selenium are associated with an increased risk of various malignancies, including colorectal cancer [10,11]. Interestingly, clinical studies show that low serum concentrations are associated with elevated levels of this element in tumour-transformed colorectal tissues [10]. Nevertheless, Se’s role in cancer prevention and the colon is still not fully understood, and the epidemiological data is inconsistent [3,4,12,13,14,15].

Zn is a critical component of various metalloenzymes, including cytoplasmic enzymes (for instance, superoxide dismutase and phosphodiesterase), mitochondrial enzymes (such as cytochrome oxidase and pyruvate carboxylase), nuclear enzymes like DNA and RNA polymerases, and enzymes of the Golgi apparatus (among other things, peptidase and mannosidase). This essential element also contributes to the regulation of gene expression, thus affecting immunity, apoptosis, and the prevention of oxidative stress. Zinc ions are integral to structural and regulatory proteins, particularly transcription factors, forming ‘zinc fingers’—specialized sequences that enable transcription factors to bind to DNA and regulate gene expression [16,17]. Zinc finger proteins play critical roles in inflammation, DNA methylation, regulating cell proliferation, and invasion and metastasis in colorectal cancer. By binding specific DNA sequences, these proteins regulate gene expression, influencing cancer progression. Several zinc finger proteins, such as CIZ1, ZNF217, ZNF281, ZKSCAN3, and ZFP36, are closely linked to colon cancer progression, correlating with tumour size, lymph node metastasis, and TNM stage [18,19,20,21,22]. Moreover, Zn deficiency impairs DNA repair by compromising the p53 protein’s function. In a study conducted on a colon cancer cell line (HCT-116), Zn-stabilized adenomatous polyposis coli (APC) protein resulted in the inhibition of cell proliferation [23]. An inverse relationship between Zn intake and many gastrointestinal malignancies, especially cancer and precancerous lesions of the colon, was observed [3,24]. However, not all of the investigators reported such associations [25].

Within the cell, Cu, as an essential cofactor of various chaperones and enzymes, plays a fundamental role in regulating cell growth and influencing gene expression through the oxidation of guanosine and adenosine residues in nucleic acids or changes in transcription and growth factor activities [26]. Moreover, Cu is critical for immune functioning. In general, intracellular levels of copper ions are low, and elevated levels can induce cytotoxicity and even lead to cell death. Recently, a new form of programmed cell death has been discovered which results from excessive copper levels. Intracellular copper enhances the degradation of Fe-S cluster proteins and proteotoxic reactions by promoting the thiooctylated aggregation process of mitochondria-associated proteins, ultimately leading to cell death, called cuproptosis [27]. It was found that inflammatory cytokines are Cu-dependent, and copper metabolism is altered in inflammation. Cu can be involved in the development and progression of malignancy through the generation of ROS, which can activate signalling pathways for cellular proliferation. On the other hand, Cu and Zn are indispensable for the structure and activity of Cu, Zn-superoxide dismutase (Cu, Zn-SOD). It was demonstrated that Cu is crucial for angiogenesis, inter alia, due to the activity of such factors as the vascular endothelial growth factor (VEGF), interleukin-1, and the basic fibroblast growth factor [28]. An increased Cu concentration in serum and tissue was detected in various cancers, including in the gastrointestinal tract, but contradictory data was also published [3,29,30].

The TAS (total antioxidant status) value indicates the plasma’s and, indirectly, the body’s overall capacity to combat oxidative stress, which is especially important in diseases with a high oxidative load, such as malignancies. As a marker of antioxidant reserves, TAS represents the combined activity of non-enzymatic antioxidants (e.g., protein thiol groups—mainly albumin, uric acid, vitamins C and E, bilirubin, and glutathione) and enzymatic antioxidants (e.g., superoxide dismutase SOD3, catalase, and glutathione peroxidase GPx3) found in plasma. These antioxidants act together to suppress oxidative processes by neutralizing ROS, which play a role in all stages of tumour development [31]. A delicate balance among many interdependent antioxidants seems to be more critical for the protective capacity of the antioxidant system than the level of a single antioxidant because TAS is a parameter that considers the dynamics of changes in the antioxidant capacity of plasma [32].

This study aimed to evaluate the clinical utility of serum Se, Zn, and Cu concentrations and plasma TAS in the diagnosis of CRA and CRC including subgroups classified as N0 (lymph nodes negative) or N+ (lymph nodes positive).

## 2. Materials and Methods

### 2.1. Patients

**Figure 1:**
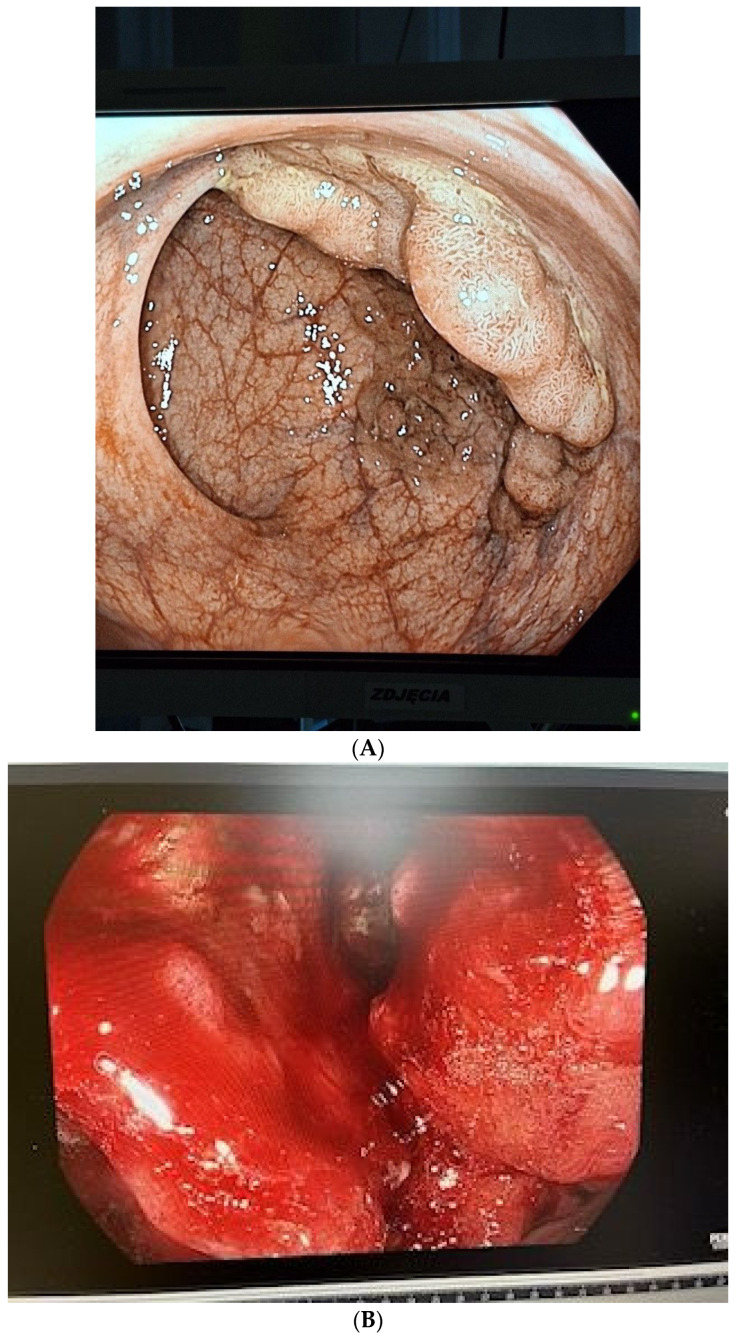
(**A**) The endoscopy image with the colorectal adenoma (CRA); (**B**) The endoscopy image with the colorectal cancer (CRC).

Study participants were recruited for two years in the Department of General, Minimally Invasive and Trauma Surgery, Franciszek Raszeja Municipal Hospital in Poznan, Poland. All individuals underwent a complete clinical examination and colonoscopy. Patients with the following conditions were excluded from the study: uncontrolled diabetes mellitus, advanced atherosclerosis of any location, any clinically evident inflammatory process, malnutrition or malabsorption, alcohol abuse, and malignancy other than colorectal cancer (based on an analysis of available medical documentation). Based on histopathology findings (H-P), if necessary, all 79 subjects were divided into three groups: with colorectal cancer (H-P: adenocarcinoma), with colorectal adenoma (H-P: adenoma), and controls (endoscopy/H-P: normal, diverticulosis, spastic colopathy, haemorrhoids) (Figure 1). The cancer progression was classified as N0 (lymph nodes negative, i.e., A and B Dukes’ stages) or N+ (lymph nodes positive, i.e., C and D Dukes’ stages).

Blood was collected after an overnight fast and prior to any treatment (chemotherapy, radiotherapy, or surgery) from individuals not taking any mineral/vitamin supplementation. Blood was obtained from an antecubital vein from all participants. Off-the-clot serum (for trace elements) and plasma samples (Li-Heparin for TAS) without haemolysis were stored at −80 °C until assayed.

This study was approved by the Bioethical Committee of the Poznan University of Medical Sciences, Poznan, Poland (protocol no 1135/04). All persons were informed of this study’s purpose and gave written informed consent.

### 2.2. Determination of Serum Se, Zn, and Cu and TAS

The Se concentrations in serum samples (20 µL) were determined by graphite furnace atomic absorption spectroscopy using a PerkinElmer Zeeman 3030 spectrometer (PerkinElmer Instruments, Norwalk, CT, USA). Standard solutions of Se were used (70 g of BSA and 9 g of NaCl per 1 L). To improve the detectability of Se in the serum, a chemical modifier was used (1 g of AgNO_3_, 2 g of Cu(NO_3_)_2_·3H_2_O, 2 g of Mg(NO_3_)_2_·3H_2_O, and 4 mL of HNO_3_ 65%) [33]. In contrast, using a PerkinElmer spectrometer, Zn and Cu levels were assayed using flame atomic absorption spectrometry. For this purpose, serum samples (0.5 mL) were diluted with deionized water (1:1 proportion). Standard solutions (Baker Chemical Co., Philipsburg, NJ, USA) were used of Zn (1000 mg/L in 0.5 mol/L HNO_3_) and Cu (1000 mg/L in 0.3 moL/L HNO_3_). For the calibration, working solutions were also used (a solution of standards and deionized water). The accuracy of element determinations was monitored using the reference serum SeronormTM (Nycomed Pharma, Oslo, Norway) [34].

Total antioxidant status (TAS) was measured by a colourimetric test (RANDOX Laboratories Ltd., Crumlin, UK) on a StatFaxTM 1904 Plus spectrometer (Awareness Technology, Inc., Palm City, FL, USA). Intra-assay coefficients of variation (CV) 1.65% and inter-assay CV 3.6% were calculated for TAS.

### 2.3. Statistical Analysis

Total antioxidant status (TAS) was measured by a colourimetric test (RANDOX Laboratories Ltd., Crumlin, UK) on a StatFaxTM 1904 Plus spectrometer (Awareness Technology, Inc., Palm City, FL, USA). Statistica 13.0 for Windows (StatSoft, Inc., Tulsa, OK, USA) was used for the statistical analysis. The results were expressed as a median value and an interquartile range. The normality of data distributions was checked using the Shapiro–Wilk test, and the homogeneity of variance was tested using Levene’s test. The multiple comparisons of mean ranks for all groups were performed using the non-parametric Kruskal–Wallis test with the subsequent Dunn test. Because of all the non-parametric data, the correlations were analysed using Spearman’s correlation coefficient (R). The significance of sex and smoker distributions was evaluated by the Fisher exact test. The MedCalc Statistical Software version 15.11.4 was used for the receiver operating characteristic (ROC) curves analysis. Calculation formulas for statistical parameters such as sensitivity and specificity are presented in a new table (Table 1).

To determine the sample size, concerning the power of the test for the difference between the null hypothesis mean and the population mean, we accepted a target power of 0.9 with a probability of 0.05 for type I error (alpha). We chose concentrations of selenium, an essential biochemical parameter, whose mean value of the null hypothesis (the control group in this study) and the level of type II error (sigma)—estimation based on the variance value—is known from the literature and the laboratory ranges determined in our study. The final sample size of patients was chosen using the above estimations. In the multiple testing, Bonferroni correction was used. *p* < 0.05 was considered statistically significant.

## 3. Results

**Figure 2:**
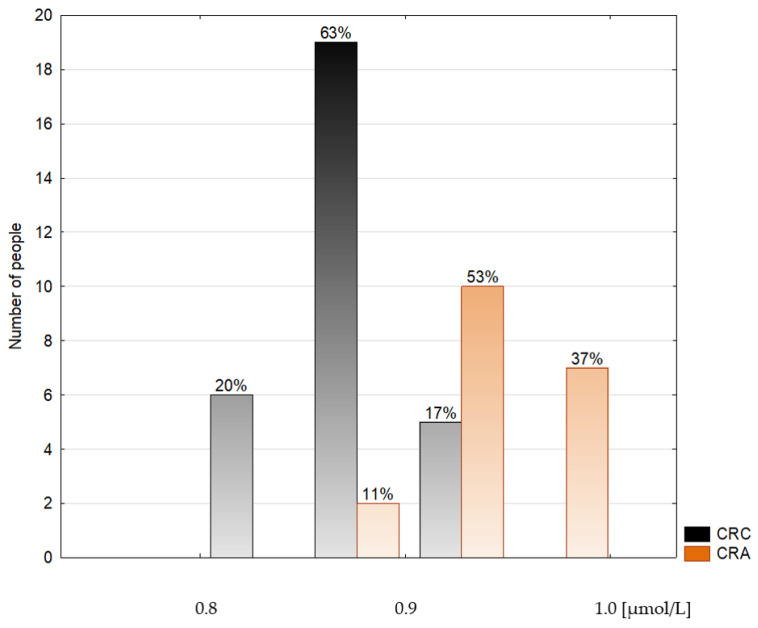
Histogram of the serum selenium concentrations in the colorectal cancer patients (CRC) and the colorectal adenoma group (CRA).

**Figure 3:**
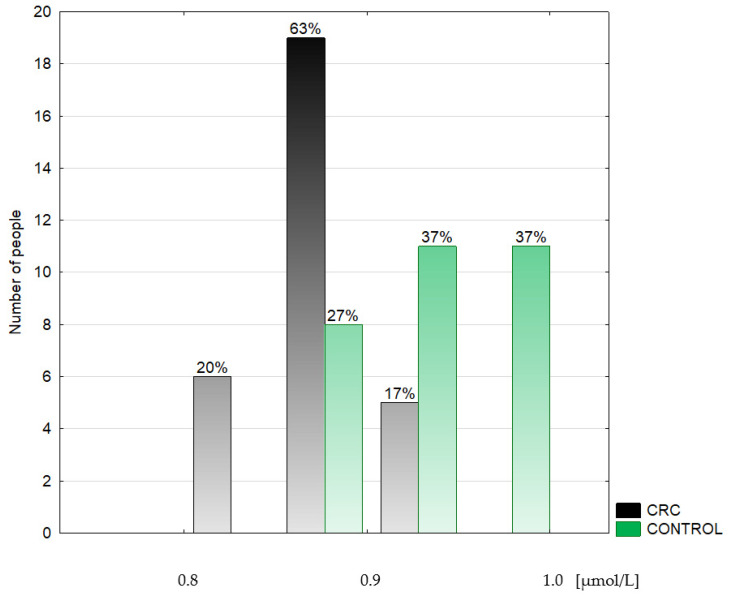
Histogram of the serum selenium concentrations in the colorectal cancer patients (CRC) and the control group (CONTROL).

**Figure 4:**
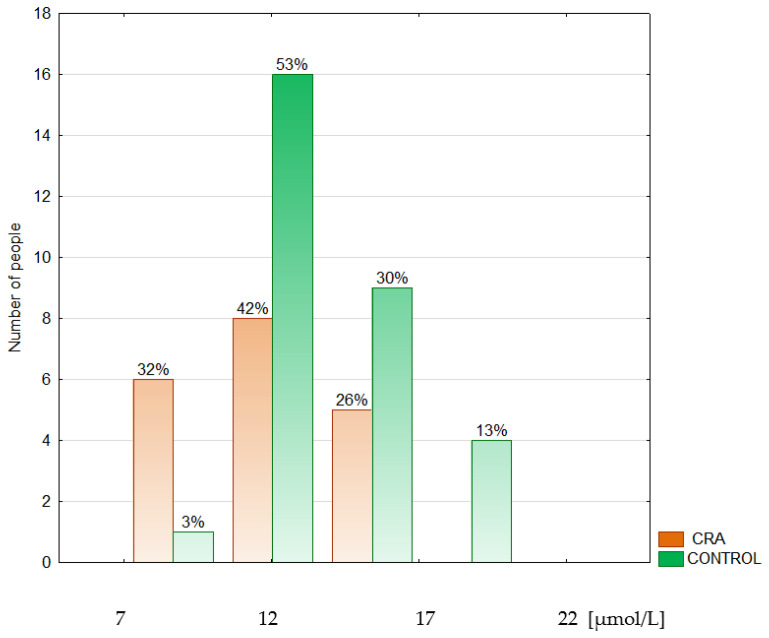
Histogram of the serum zinc concentrations in the colorectal adenoma patients (CRA) and the control group (CONTROL).

Epidemiological and clinical data are shown in Table 1. The median age of the groups studied neither differed significantly from nor correlated with any other variable. The serum Se concentration was expressed in µmol/L and µg/L. The last unit was introduced for the discussion because most other authors referred to traditional units. The conversion factor to SI units for Se is 0.1266 (µmol/L) (Table 2, Figure 2, Figure 3 and Figure 4).

The data are presented as a median and interquartile range. This study revealed a lower (by 9.1%) serum Se concentration in the CRC group (66.39 (64.58–68.51) µg/L) in comparison to both the CRA (72.45 (70.88–75.6) µg/L, *p* < 0.0001) and the CONTROL groups (71.66 (68.51–74.02) µg/L, by 7.9%, *p* < 0.0001). The difference between the CRC and the CONTROL groups was not associated with the stage of the malignancy (N0: 66.15 (64.58; 67.73) µg/L, N+: 66.86 (63.79–68.51) µg/L). The serum median Zn concentration was found to be decreased (by 17.9%) in the CRA group (11.62 (10.09–14.08) µmol/L) when compared to the CONTROL group (13.7 (13.01–15.45) µmol/L), *p* = 0.019) and did not differ between cancer subgroups (N0: 13.24 (11.78–14.54) µmol/L, N+: 12.78 (11.70–13.85) µmol/L). The serum Cu concentration did not differ significantly among the groups studied nor cancer subgroups. Still, the tendency to maintain a higher level of Cu in the CRC group was observed compared to both the CRA and the CONTROL groups. Plasma TAS was significantly lower (by 27.8%) in the CRC group (1.15 (1.09; 1.41) mmol/L, *p* = 0.017) than in the CONTROL group (1.47 (1.29; 1.59) mmol/L) (Table 3).

The median TAS concentration was similar in both cancer subgroups. Analysis of the relationships between serum trace elements and plasma TAS revealed a significant, positive correlation between Se and TAS in the CRC and the CONTROL groups. Moreover, an inverse correlation was found between Cu and TAS in the CONTROL group. No correlation was observed in the CRA group. Zn did not correlate with any parameters studied in a given population. Detailed data have been presented in Table 4.

**Figure 5:**
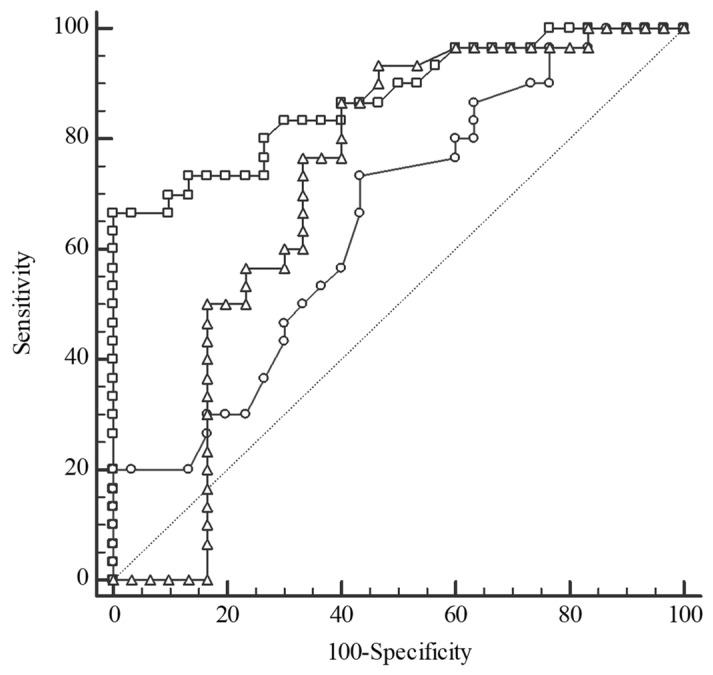
Receiver operating characteristic (ROC) curves for selenium (Se) level (square), total antioxidant status (triangle), and zinc (Zn) level (circle) in the differentiation between colorectal cancer patients (CRC) and the control group (CONTROL). Selenium: cut-off value ≤ 69.3 µg/L, sensitivity 100%, specificity 66.67%, AUC 0.873, 95%CI: 0.761–0.945, *p* < 0.0001; Total antioxidant status: cut-off value > 1.15 mmol/L, sensitivity 93.33%, specificity 53.33%, AUC 0.718, 95%CI: 0.587–0.827, *p* = 0.002; Zinc: cut-off value > 13.01 µmol/L, sensitivity 73.33%, specificity 56.67%, AUC 0.652, 95%CI: 0.518–0.771, *p* = 0.032; AUC—area under the ROC curve.

For differentiation of the CRC patients from the CONTROL, the serum Se concentration was indicated by AUC (area under ROC curve) as a good test, whereas plasma TAS and Zn levels were a fair and a poor test, respectively (Figure 5).

**Figure 6:**
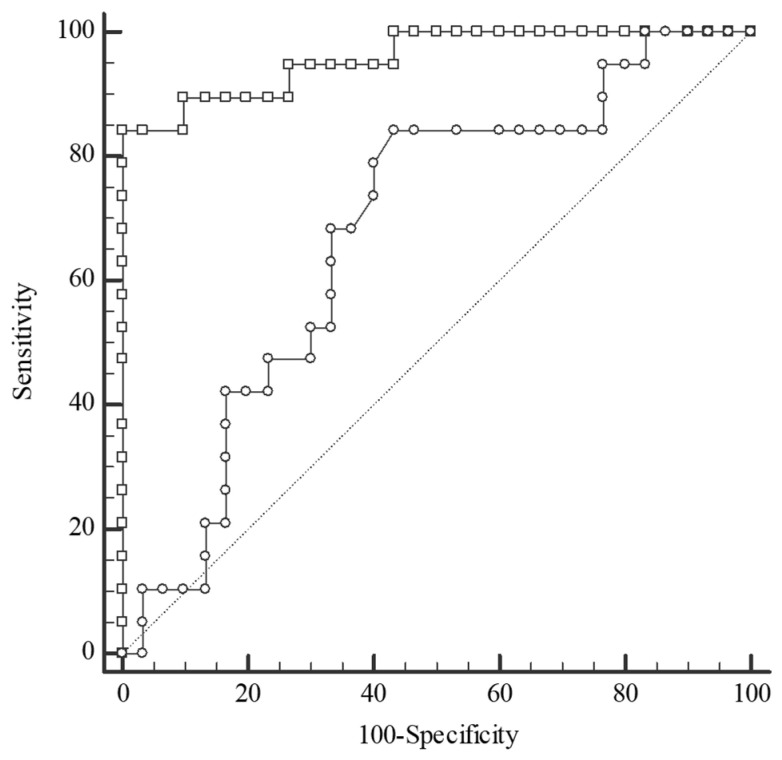
Receiver operating characteristic (ROC) curves for selenium (Se) level (square) and total antioxidant status (circle) in the differentiation between groups: colorectal cancer (CRC) and colorectal adenoma (CRA). Selenium: cut-off value > 69.3 µg/L, sensitivity 84.21%, specificity 100%, AUC 0.958, 95%CI: 0.858–0.995, *p* < 0.0001; Total antioxidant status: cut-off value > 1.19 mmol/L, sensitivity 84.21%, specificity 56.67%, AUC 0.679, 95%CI: 0.530–0.805, *p* = 0.024; AUC—area under the ROC curve.

**Figure 7:**
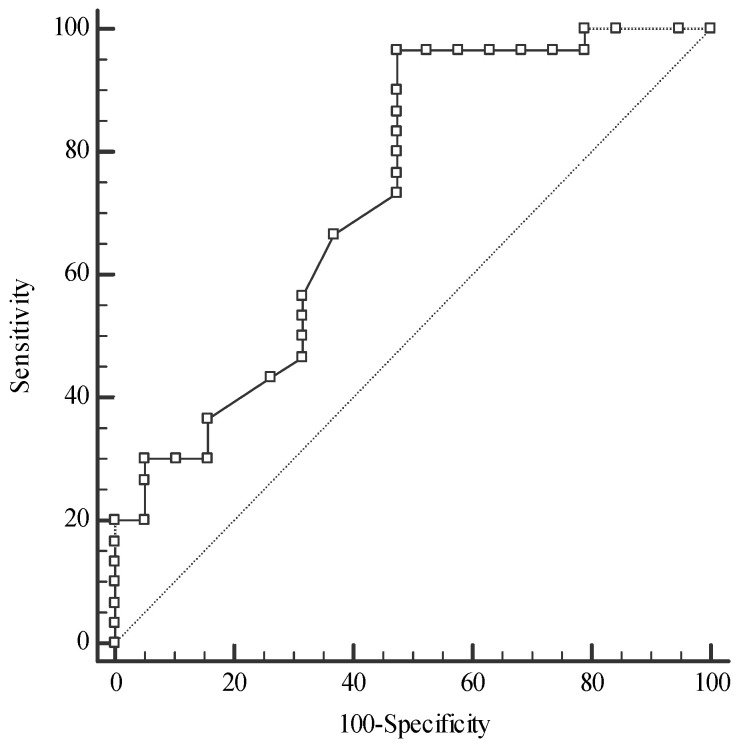
Receiver operating characteristic (ROC) curves for zinc concentration in the differentiation of colorectal adenoma patients (CRA) from the control group (CONTROL); Zinc: cut-off value > 11.62 µmol/L, sensitivity 96.67%, specificity 52.63%, AUC 0.730, 95%CI: 0.584–0.847, *p* = 0.003; AUC—area under an ROC curve.

For differentiation between the CRC and the CRA groups, serum Se and plasma TAS concentrations were of excellent and fair accuracy, respectively (Figure 6). ROC curve analysis revealed that the Se level was of the highest diagnostic utility for the discrimination of the CRC group from both the CRA (AUC 0.958, sensitivity 84.21%, specificity 100%) and CONTROL groups (AUC 0.873, sensitivity 100%, specificity 66.67%), with a cut-off value > 69.3 µg/L. The serum Zn concentration can help to distinguish the CRA group from the CONTROL group with reasonable accuracy (Figure 7).

## 4. Discussion

The geographical Se distribution in the soil varies significantly between regions of low Se content (e.g., Europe) and regions rich in Se (e.g., the USA), resulting in significant differences in daily intake among populations (the estimated average Se daily intake is 25–100 µg in Europe and 90–134 µg in the USA). Hence, the plasma Se concentration also varies significantly among populations (a mean of 80 µg/L in Europe and 136 µg/L in the USA) and individuals. The risk-benefit window for Se intake and plasma Se level seems narrow. These facts lead to conflicting results from epidemiological and interventional studies, but also to the conclusion that supplementation can be beneficial only if the micronutrient status is inadequate [5,36]. The plasma Se concentration is defined as a short-term marker of Se status, reflecting the most recent intake of the micronutrient. The activity of extracellular selenoproteins GPx and SeP as biomarkers is preferable to assess Se status and the adequacy of Se long-term intake. The maximum plasma GPx3 activity is achieved when the plasma Se concentration is 90–98 µg/L, and the plasma SeP concentration reaches its plateau at plasma Se level 124 µg/L [36]. Based on European epidemiological and interventional studies, the optimal plasma Se concentration is proposed to be 100–120 µg/L. At this Se level, cancer morbidity and mortality were a few times lower [37,38].

**Figure 8:**
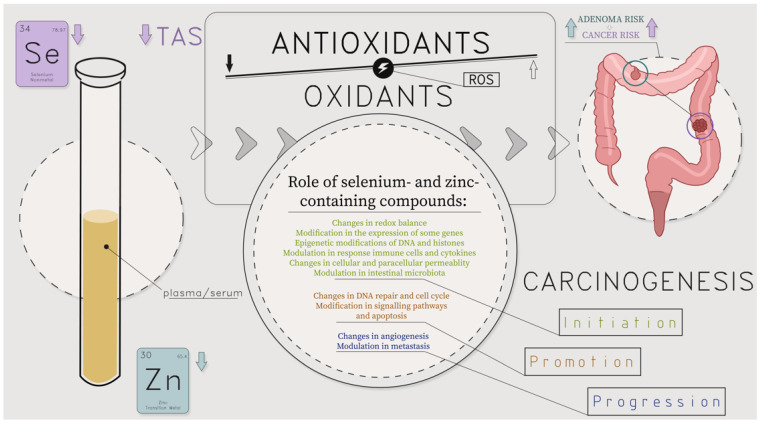
Molecular and cellular influence of lower levels of selenium and selenium-containing compound families: glutathione peroxidases, selenoprotein P, and lower levels of zinc and zinc-containing compound families: metalloenzymes, including cytoplasmic enzymes (for instance, superoxide dismutase and phosphodiesterase), mitochondrial enzymes (such as cytochrome oxidase and pyruvate carboxylase), nuclear enzymes like DNA and RNA polymerases on colorectal polyps (adenoma, CRA) and colorectal cancer (CRC) development; ROS—reactive oxygen species; TAS—total antioxidant status; authors’ own compilation based on [3,5,6,16,17,18,19,20,21,22,53,54,55,56,57,58,60,61,65,66,67,68,69,77,78,79,80,81].

A meta-analysis of 49 prospective studies demonstrated a 30% lower risk of cancer incidence at the highest Se status compared to the lowest [39]. There is strong evidence that a low Se status may contribute to the risk of colorectal cancer development. The European prospective investigation revealed an inverse correlation between colorectal cancer risk and a higher serum Se concentration [40]. Furthermore, the association between higher Se status and the reduction of cancer risk for the colorectal, larynx, stomach, pancreas, lung, prostate, and bladder was observed [13,38,41]. In a Polish-Estonian study, a more than 13-fold increase in colorectal cancer risk was observed in persons with serum Se < 40 µg/L than in those with serum Se > 72 µg/L [42]. Similarly, in the present investigation, the serum Se concentration ranged from 60.48–76.62 µg/L, whereas the median Se in the CRC group was significantly lower than in the CONTROL and the CRA groups (see Figure 8). Moreover, in the study population, the Se level 69.3 µg/L was established to discriminate the CRC patients from the CONTROL (AUC 0.873, 100% sensitivity, and 66.67% specificity) and the CRA group (AUC 0.958, 84.21% sensitivity, and 100% specificity). An AUC value characterizes most of the recognized diagnostic markers used in clinical studies according to the ROC curve between 0.8–0.9; with values that are 0.7 and above, the predictor is deemed satisfactory [43]. Thus, we postulate that the serum Se concentration can be used in practice as a significant risk factor for colorectal cancer. In this case, people should be strongly advised to undergo a colonoscopy, take screening tests for other cancers, and start adequate Se supplementation. Such an attitude was successfully practised in the case of another cancer preventive program. Only people with a low Se level (<75 µg/L) were invited to participate in a lung cancer screening program (CT scan), resulting in a doubled lung cancer detection rate [44].

The findings of most studies suggest a protective effect of Se for colorectal adenomas. Reid et al. demonstrated a reduced risk of colorectal polyps among individuals taking Se supplementation because of the low baseline selenium level [45]. A Mendelian randomization study explored the relationship between several trace nutrients in the human organism (calcium, selenium, magnesium, phosphorus, folate, vitamins B6, B12, C, D, β-carotene, iron, zinc, and copper) and the genetic susceptibility to colorectal polyps in Europeans and found a correlation between higher selenium and β-carotene levels and lower incidence of colorectal polyps [46]. The present study found no difference in the Se concentration between the CRC and the CRA groups. To some extent, the discrepancy might result from the small group of patients with colorectal polyps. Moreover, optimal Se can be different for a particular population or country. In the population studied, serum Se concentrations were far below the optimal; thus, the possible protective effect for colonic mucosa of higher Se status within that range might not be seen. In the USA, the serum Se concentration associated with the lowest risk of colorectal adenomas was >150 µg/L. This suggests different environmental and genetic factors affecting colonic mucosa in various world regions [44]. Nevertheless, persons with colorectal adenoma would benefit from monitored Se supplementation to prevent polyp recurrence or progression to colorectal cancer.

The most frequent is mild to marginal Zn deficiency occurring without apparent symptoms [47]. Suboptimal zinc status has been recognized as a worldwide problem affecting developed and developing countries [48]. The daily zinc intake in elderly subjects in the Western world, including the USA, is only 8–10 mg, whereas the RDA (Recommended Dietary Allowance) is 15 mg [49]. In a meta-analysis, considerable variations in the mean plasma Zn level were reported: 8.5 µmol/L in Nepal, 10.44 µmol/L in New Zealand, and 17.48 µmol/L in Spain [50], which, in our opinion, can generate discrepancies in the results of studies investigating the Zn–cancer relationship in different populations. Approximately 95% of the body’s Zn is located within cells, and the cellular Zn concentration is physiologically maintained within a narrow range by a complex regulatory system. Plasma is a small Zn pool that is vulnerable to insufficient intake. Plasma Zn is used for tissue redistribution during metabolic conditions, such as trauma, infection, stress, steroid use, and after a meal. Hence, redistribution confounds the level interpretation of low plasma/serum Zn level. Biomarkers of metabolic zinc redistribution (e.g., plasma and erythrocyte metallothionein) could help to discriminate poor nutrition from the plasma/serum low Zn concentration secondary to a given metabolic condition [51].

Zinc deficiency can be considered a risk factor for cancer development [13,52,53,54]. Zinc finger proteins are expressed infrequently in the normal colonic mucosa. Still, they are expressed in more than 80% of colorectal cancers, suggesting that dysregulation of zinc finger proteins may be implicated in colon carcinogenesis. At the molecular level, metals such as iron can substitute for zinc. They may be responsible for metal-induced DNA damage and subsequent carcinogenesis, suggesting a close relationship between the two nutrients. Lee et al. examined the association between colorectal cancer incidence and dietary intake of zinc, heme iron, and alcohol during 15 years of follow-up. Dietary zinc intake was reported to be associated with a decreased risk of distal colorectal cancer, regardless of iron or alcohol consumption [54]. A meta-analysis of almost 20 studies found that the level of Zn intake was inversely correlated with colorectal cancer [55]. Moreover, the recent prospective observational study reported a correlation between low serum zinc levels and poor prognosis in the cases of patients with stages I–III of colorectal cancer, connected with the percentage of distant metastasis and survival [53]. However, in another meta-analysis, variations in serum and tissue zinc levels in colorectal cancer were documented [56]. In epidemiological studies, Zn appears protective against cancer when Zn-deficient individuals are compared to Zn-sufficient groups [57,58].

In the present study, serum median Zn concentrations in the CRC and CONTROL groups were within the reference values but close to the lower reference limit (12–18 µmol/L) [59], whereas in the CRA group, the median Zn was borderline. The investigation revealed a decreased serum Zn concentration in the CRA group compared to the CONTROL group (see Figure 8). A trend towards lower Zn levels was observed in the CRC group (advanced stage) compared to the CRA and CONTROL groups. Our previous study found a lower serum Zn concentration in colorectal cancer and polyp patients than in the controls; considering the advanced state of the malignancy, the difference was observed only between the cancer subgroup with metastases and the controls [60]. Similarly, Gupta et al. showed a lower serum Zn concentration only in advanced CRC patients compared to the controls and a higher Zn level in the colorectal cancer tissue than in the normal mucosa [61]. In the population studied, a serum Zn level of 11.62 µmol/L was established to discriminate the CRA group from the CONTROL group (with 96.67% sensitivity and 52.63% specificity), suggesting that the Zn level can be used in clinical practice for the management of colorectal adenoma.

A copper-lowering therapy for Cu overload disorder was proven to limit tumour angiogenesis [62]. This corresponds with reports indicating that the serum Cu concentration correlates with tumour incidence and malignant progression and recurrence in many cancers, including colorectal. The mean serum Cu level was reported to be increased in patients with colorectal cancer in comparison to healthy controls [29,62]. Maintaining copper balance in cancer cells involves the complex interaction of several factors. The reasons for the increased copper content in tumours may be twofold. First, cancers, especially those rapidly growing, have high metabolic requirements. It should be noted that copper is a cofactor for many enzymes involved in cellular energy metabolism, such as cytochrome c oxidase, and in antioxidant defence via, for example, SOD. Therefore, the requirement for copper in these processes may be increased in cancer cells. Secondly, upregulation of the copper transporter protein (CTR1) has been observed in tissues affected by hypoxia, which is associated with cancer. Hypoxia-inducible factor 1-alpha (HIF-1α) can indirectly stabilize and activate the transcription of many genes involved in copper metabolism, including those controlling CTR1, thereby contributing to higher copper levels in cancer cells. Indeed, CTR1 expression is increased in various cancers [63].

On the other hand, Cu serum concentration was observed to be lower in Iranian colorectal cancer patients than in healthy controls [30]. These discrepancies can partly be explained by the observation that decreased and increased Cu intake can be linked to colorectal cancer development. Chronic Cu exposure and overload were associated with oxidative damage. On the other hand, deficiency in dietary Cu also influenced cellular susceptibility to oxidative damage [64]. In the present study, we demonstrated a trend only for a higher serum Cu concentration in the CRC group compared to the CONTROL group. However, Cu levels determined in the population studied were within the reference range (10.99–24.33 μmol/L) [59].

Colorectal carcinoma is characterized by oxidative stress and antioxidant imbalance. The progression of the disease is followed by an increasing intensity of redox imbalance [65,66,67]. Blood is central in modulating redox balance, as it transports and redistributes antioxidants to every body part. Thus, plasma redox status, the sum of the reducing and oxidizing equivalents in the biological fluid, results from the interaction of many different compounds and systemic metabolic processes. Coordination among various antioxidants provides excellent protection against free radical injury compared to any compound alone [68]. The body fluids’ antioxidant activity is directly related to the levels of antioxidants and free radical production, and it is under substantial genetic control (Figure 8), [69]. Evidence regarding the association between TAS (determined by the same method as in the present work) and cancer is limited [67,70,71,72]. An Italian case-control study found an inverse relationship between dietary TAS and colorectal cancer risk (see Figure 8) [70]. In another study conducted before treatment, the serum total oxidant status was increased, whereas the TAS level was decreased in colorectal cancer patients compared to the healthy controls [67,71]. An increased serum TAS concentration was also associated with reduced breast cancer risk [72].

In this investigation, the plasma TAS level in the CONTROL group was similar to the TAS level determined using the same method in the healthy volunteers (1.48 ± 0.41 mmol/L) also recruited from the Polish population, and it was within the reference range for Europeans (1.30–1.77 mmol/L) [73]. We found a significantly decreased TAS level in the CRC group compared to the CONTROL group, and the significance of the difference was maintained in patients with advanced malignancy (N+). The plasma TAS level of 1.15 mmol/L was established to differentiate between the CRC group and normal conditions with 93.33% sensitivity and 53.33% specificity. In our opinion, a lower median TAS level in cancer patients might suggest the exhaustion of the plasma antioxidant system. It can result from oxidative stress during colorectal malignancy, as it was frequently reported [71,74,75]. A recent study suggests that higher exposure to antioxidants, assessed through an oxidative balance score, may decrease the occurrence of CRC and its metastases in Europeans [76]. There remains a need to investigate the extent to which a decreased TAS level might be the reason for and the consequence of the malignant process.

In the present study, the Se concentration correlated positively with TAS in all groups of patients except for the CRA subjects. Hence, the Se level contributes to the antioxidant defence, and therefore, monitoring its serum level can be included in the complex prevention of oxidative stress-related diseases, such as colorectal cancer. Al-Ansari et al. also observed a positive correlation between serum glutathione peroxidase activity and Se concentration [3]. On the other hand, in our study, the Se level was negatively associated with Cu in all patients and the CONTROL group. Zn did not correlate with any parameters studied in a given population. Similarly, Al-Ansari et al. showed no correlation between the Zn level (and also Cu concentration) and serum glutathione peroxidase activity [3].

Low concentrations of Se in serum in the CRC group and Zn in the CRA group were observed, which might suggest the existence of different distribution mechanisms for these microelements (in the blood and the intestines) (see Figure 8). Kucharzewski et al. found a higher content of Se and Zn in the colon tissues of the CRC group than in the CRA group [77]. In studying these elements in the tissues of control (normal colon and rectal) and CRA patients, only Zn levels were present in similar concentrations. The level of Se was higher in CRA groups vs. controls [78].

Our study group was recruited from a population with a relatively low zinc serum status. Biomarkers of metabolic Zn redistribution (e.g., plasma and erythrocyte metallothionein) could help to discriminate poor nutrition from the plasma/serum low Zn concentration secondary to a given metabolic condition [51]. Zn influences the regulation of intestinal immune cell function and gut microbiota composition and contributes to the stability of the intestinal mucosal chemical barrier [79]. It was found that the development of precancerous lesions in the colon was associated with decreased levels of Zn in plasma and tissue and the activity of zinc-related enzymes, and these biochemical markers further decreased with the progression to carcinoma in rats with chemically induced colon cancer [80]. Moreover, Zn affects DNA transcription and replication through zinc finger proteins and the p53 tumour suppressor protein. Therefore, Zn deficiency can influence the expression of numerous transcription factors, e.g., AP-1, NF-κB being involved in carcinogenesis, including of the colon [79,81]. It is not known to what extent changes in Se and Zn concentrations are the cause or effect of the ongoing pathology of the colonic mucosa (Figure 8). Further investigation is required to establish whether these relationships refer to the primary dysregulation of Se, Zn, Cu, and TAS homeostasis and their mutual interactions already noticeable at early stages of colonic carcinogenesis or whether they are secondary to cancer-related inflammation.

The strengths of our study include the comparison of patients with colon polyps or cancer to the control group so the three groups can mimic the natural history of the development of colorectal cancer through the state of precancerous lesions. All participants underwent a colonoscopy. This study comprised an analysis of micronutrients (Se, Zn, and Cu) and plasma TAS, which can give better insight into the complex relations among many factors affecting the individual risk of colorectal cancer development. Despite this, a limitation of our study is the relatively small size of the groups. The final sample size resulted from applying the exclusion criteria, i.e., pathological conditions and mineral/vitamin supplementation, possibly influencing the results of blood tests performed in this study. Additionally, we were not able to follow the same persons over many years to observe the influence of plasma Se, Zn, Cu, and antioxidant status, among other factors, on the malignant process in colonic mucosa.

Future investigations should consider other confounding factors influencing redox and micronutrient status and the risk of colorectal cancer, such as alcohol intake, physical activity, and various supplements, including probiotics, diet, genotypes, and available molecular aspects of carcinogenesis. Likewise, research should include a marker of oxidative damage and a marker of antioxidant defence (e.g., plasma TAS) to obtain a comprehensive insight into the oxidant-antioxidant status during colorectal cancer development.

## 5. Conclusions

An individualised risk of colorectal adenoma and cancer approach could be comprised of detecting plasma Se, Zn, and TAS values, but monitoring plasma Cu concentration seems to be not useful. It would meaningfully contribute to personalised medicine oriented on the multifactorial chronic civilization-linked diseases, such as colorectal cancer. Comprehensive, intensive primary and secondary prevention are the most humane and cost-effective.

## Figures and Tables

**Table 1:** Calculation formula for sensitivity, specificity, and 95% CI (for instance, for colorectal cancer (CRC)).

Parameters/Formula	CRC Present	CRC Absent
Index Test Positive	True positive (TP)	False positive (FP)
Index Test Negative	False negative (FN)	True negative (TN)
Sensitivity = TP/(TP + FN) Specificity = TN/(TN + FP) 95% CI of AUC = AUC ± 1.96 SEM		

95% CI of AUC—95% confidence interval in AUC (area under ROC curve) value; SEM—standard error of the mean. Authors’ own compilation based on [35].

**Table 2:** The characteristics of the study groups. Serum Se, Zn, and Cu and plasma total antioxidant status (TAS) in the colorectal cancer (CRC) group, the colorectal adenoma (CRA) group, and controls (CONTROL).

Study Group /Parameters	CRC (n = 30)	CRA (n = 19)	CONTROL (n = 30)	*p*
Age (years)	62.5 (55.0–70.0)	62.0 (51.0–63.0)	56.5 (53.0–62.0)	NS ^#^
Gender (male/female)	16/14	7/12	3/17	NS ^#^
Smokers (n (%))	7 (23%)	4 (21%)	5 (17%)	NS ^#^
Se				
[µmol/L]	0.84 (0.82–0.87)	0.92 (0.90–0.96)	0.91 (0.87–0.94)	*p* < 0.0001 (CRC vs. CRA)
[µg/L]	66.39 (64.58–68.51)	72.45 (70.88–75.6)	71.66 (68.51–74.02)	*p* < 0.0001 (CRC vs. CONTROL) NS (CRA vs. CONTROL)
Zn [µmol/L]	13.01 (11.78–14.38)	11.62 (10.09–14.08)	13.7 (13.01–15.45)	NS (CRC vs. CRA) NS (CRC vs. CONTROL) *p* = 0.019 (CRA vs. CONTROL)
Cu [µmol/L]	17.86 (14.64–21.88)	16.85 (14.96–18.57)	16.13 (13.69–19.52)	NS ^#^
TAS [mmol/L]	1.15 (1.09–1.37)	1.36 (1.25–1.76)	1.47 (1.29–1.59)	NS (CRC vs. CRA) *p* = 0.017 (CRC vs. CONTROL) NS (CRA vs. CONTROL)

Data are presented as a median (interquartile range); n—number of people; Se—selenium; Zn—zinc; Cu—copper; *p*—level of statistical significance in Dunn test (in case of numerical data) and Fisher exact test (non-numerical data); M—male; F—female; NS—statistically insignificant difference; NS ^#^—statistically insignificant difference: CRC vs. CRA, CRC vs. CONTROL and CRA vs. CONTROL.

**Table 3:** Serum Se, Zn, and Cu and plasma total antioxidant status (TAS) in the colorectal cancer (CRC) group in relation to the progress of the disease: N0 (lymph nodes negative, i.e., A and B Dukes’ stages) or N+ (lymph nodes positive, i.e., C and D Dukes’ stages) in comparison to the colorectal adenoma (CRA) group and controls (CONTROL).

Study Group /Parameters	CRC N0 (n = 18)	CRC N+ (n = 12)	*p*
Age (years)	64.5 (59.0–70.0)	59.0 (55.0–70.0)	NS ^##^
Gender (male/female)	6/12	10/2	NS ^##^
Smokers (n (%))	3 (17%)	4 (33%)	NS ^##^
Se [µmol/L] [µg/L]	0.84 (0.82–0.86) 66.15 (64.58–67.73)	0.85 (0.81–0.87) 66.86 (63.79–68.51)	*p* < 0.0001 (CRC N0 vs. CRA) *p* = 0.0005 (CRC N0 vs. CONTROL) *p* = 0.0005 (CRC N+ vs. CRA) *p* = 0.006 (CRC N+ vs. CONTROL)
Zn [µmol/L]	13.24 (11.78–14.54)	12.78 (11.70–13.85)	NS ^##^
Cu [µmol/L]	18.97 (15.74–22.04)	17.40 (14.25–19.76)	NS ^##^
TAS [mmol/L]	1.15 (0.98–1.37)	1.15 (1.09–1.41)	NS ^##^

Data are presented as a median (interquartile range); n—number of people; Se—selenium; Zn—zinc; Cu—copper; *p*—level of statistical significance in Dunn test (in case of numerical data) and Fisher exact test (non-numerical data); M—males; F—females; NS—statistically insignificant difference; NS ^##^—statistically insignificant difference: CRC N0 vs. CRA, CRC N0 vs. CONTROL, CRC N+ vs. CRA and CRC N+ vs. CONTROL.

**Table 4:** Statistically significant correlations between serum Se, Cu, and plasma total antioxidant status (TAS) in all patients studied and in the groups of patients: colorectal cancer (CRC), colorectal adenoma (CRA), controls (CONTROL).

Study Group /Parameters	All Patients (n = 79)	CRC (n = 30)	CRA (n = 19)	CONTROL (n = 30)
Se vs. TAS	R = 0.473 *p* < 0.0001	R = 0.383 *p* = 0.037	NS	R = 0.432 *p* = 0.017
Se vs. Cu	R = −0.409 *p* < 0.0001	NS	NS	R= −0.527 *p* = 0.003

Se—selenium; Cu—copper; R—Spearman’s correlation coefficient; n—number of people; *p*—level of statistical significance in Spearman’s test; NS—statistically insignificant difference.

## Data Availability

Data are contained within the article.
